# Enhancement of cognitive function in mice with Alzheimer’s disease through hyperbaric oxygen-induced activation of cellular autophagy

**DOI:** 10.3389/fnagi.2024.1418081

**Published:** 2024-09-25

**Authors:** Qian-Qian Fan, Yong-Min Chen, Yong-Sen Fu, Xiao-Shan Li, Ji Zeng, Shao-Zhen Bian, Bin-Bin Li, Zhen-Hua Song

**Affiliations:** ^1^Department of Rehabilitation Medicine, The Affiliated Haikou Hospital of Xiangya Medical College, Central South University, Haikou, Hainan, China; ^2^Department of Functional Diagnosis, The Second Affiliated Hospital of Hainan Medical University, Haikou, Hainan, China; ^3^Department of Pediatric, Haikou Hospital of the Maternal and Child Health Hospital, Haikou, Hainan, China

**Keywords:** Alzheimer’s disease, APP/PSl double-transgenic mice, autophagy, hyperbaric oxygen, mTOR, PI3K

## Abstract

**Objective:**

In this study, we examined the effectiveness of hyperbaric oxygen (HBO) therapy in ameliorating cognitive deficits in mice with Alzheimer’s disease (AD), while also assessing its impact on the autophagic pathway within the context of AD.

**Methods:**

20 double-transgenic mice expressing the amyloid precursor protein and presenilin 1 (APP/PS1) were purposefully selected and randomly assigned to groups A and B. Concurrently, 20 C57BL/6 mice were chosen and randomly categorized into groups C and D, each consisting of 10 mice. Mice in groups B and D received HBO treatment. The Morris water maze assay was used to assess changes in mouse behavior. Immunohistochemistry techniques were used to quantify the expression levels of amyloid-beta 42 (Aβ42) and microtubule-associated protein 1A/1B-light chain 3 (LC3) in hippocampal tissues, while western blot analysis was used to investigate the levels of LC3-II, p62, phosphoinositide 3-kinase (PI3K), and mammalian target of rapamycin (mTOR) proteins within hippocampal tissues.

**Results:**

Mice allocated to group B exhibited reduced escape latency and prolonged dwell time in the target quadrant compared to other groups. Histological examination revealed conspicuous plaque-like deposits of Aβ42 in the hippocampal tissues of mice in groups A and B. Group B displayed diminished Aβ42-positive reactants and augmented microtubule-associated protein 1A/1B-LC3-positive reactants compared to group A. LC3-positive reactants were also detected in the hippocampal tissues of mice in groups C and D, surpassing the levels observed in groups A and B. Furthermore, group B demonstrated significantly lower expression of mTOR protein and markedly higher expression of LC3-II protein in mouse hippocampal tissues when compared to group A (*P* < 0.05). Conversely, there were no significant disparities noted in PI3K and p62 protein expression between groups B and A. Notably, no discernible discrepancies were observed in the expression levels of mTOR, PI3K, LC3-II, and p62 proteins between groups C and D within mouse hippocampal tissues.

**Conclusion:**

HBO treatment demonstrates efficacy in enhancing cognitive function in mice with AD and holds promise as a potential therapeutic intervention for AD by facilitating the activation of the mTOR pathway-mediated autophagy.

## 1 Introduction

Alzheimer’s disease (AD) represents a prevalent neurodegenerative disorder and is the leading cause of dementia globally. The Special Report examines the patient journey from awareness of cognitive changes to potential treatment with drugs that change the underlying biology of Alzheimer’s. An estimated 6.7 million Americans age 65 and older are living with Alzheimer’s dementia today. This number could grow to 13.8 million by 2060 barring the development of medical breakthroughs to prevent, slow or cure AD (Alzheimer’s Association Report, 2023). The neuropathological hallmarks of AD primarily encompass the formation of senile plaques (SPs) resulting from extracellular deposition of β-amyloid (Aβ), the development of neurofibrillary tangles induced by aberrant phosphorylation of intracellular tau proteins, and the degeneration of cholinergic neurons, processes modulated by a diverse array of factors including oxidative stress, inflammatory responses, cellular apoptosis, and cell autophagy. Throughout the progression of AD, changes in Aβ levels, neuronal survival and apoptosis, as well as the restoration of cognitive memory function, are intricately linked with the regulation of autophagy, with dysfunction of autolysosomes emerging in the early stages of AD in most patients (Zhao et al., 2020; de la Cueva et al., 2022). The interplay between autophagy and Aβ and tau proteins may represent a pivotal mechanism contributing to the etiology or advancement of AD. Notably, neuroinflammation and aberrant autophagic processes manifest within the AD brain prior to the onset of any discernible clinical symptoms, thereby underscoring the potential significance of autophagy in the pathogenesis of AD and its potential as a new therapeutic target. However, further investigation is warranted to elucidate the intricate mechanisms underlying these associations.

AD not only poses a significant threat to the physical and mental well-being of affected patients but also imposes a substantial burden on families and society, thereby presenting a critical challenge to public health. Compounded by the lack of effective clinical treatment options, patients diagnosed with AD often face a bleak prognosis. Non-pharmacological interventions serve as valuable adjuncts to pharmacotherapy due to their ease of implementation and reduced incidence of side effects. Hyperbaric oxygen (HBO) therapy holds promise in this regard, as it has been shown to mitigate oxidative stress, enhance oxygen diffusion distance, elevate brain tissue oxygenation, stimulate cellular proliferation, optimize cellular function, expedite capillary regeneration, and augment blood supply, thereby bolstering neuronal function and conferring beneficial conditioning and therapeutic effects on the organism. Over the past few years, the clinical efficacy and underlying mechanisms of HBO in AD treatment have been intensively studied. Both clinical and experimental trials have demonstrated the capacity of HBO to ameliorate clinical symptoms and mitigate pathophysiological abnormalities in AD murine models (Shapira et al., 2018; Chen et al., 2020; Wang et al., 2022). However, the precise mechanistic underpinnings remain elusive. Notably, autophagy, a cellular process responsive to various external stimuli, has garnered attention for its potential role in mediating the therapeutic effects of HBO. Activation of the autophagic pathway facilitates the removal of detrimental intracellular organelles and pathogens, thereby bolstering cellular resilience to hypoxia and conferring a protective effect. Based on current evidence, it is indicated that autophagy may play a pivotal role in the context of HBO-mediated AD treatment, although further elucidation is warranted.

Therefore, the objective of this study was to investigate the efficacy of HBO therapy in enhancing cognitive function in mice with AD, while also assessing its impact on the autophagy pathway within the AD context. Specifically, AD-afflicted mice underwent HBO treatment, after which cognitive function assessments were conducted. Subsequently, autophagy-related proteins, such as LC3 and p62, were analyzed to elucidate the role of autophagy in HBO-mediated AD treatment. Furthermore, the expression levels of phosphatidylinositol-3-kinase (PI3K) and mammalian target of rapamycin (mTOR) proteins were examined to clarify the influence of HBO on PI3K/AKT/mTOR pathway-mediated autophagy in mice with AD. This study therefore contributes to establishing a scientific and logical foundation for delving into the molecular mechanisms underlying HBO treatment in AD. Additionally, it offers innovative insights for the diagnosis and treatment of AD, with the objective to mitigate its progression and prevent its onset.

## 2 Materials and methods

### 2.1 Experimental animals

The APP/PS1 double-transgenic mouse model of AD was procured from Viewsolid (Beijing, China). The APPswe mutation, which involves the substitution of Lys and Met by Asn and Leu at sites 670 and 671 of the APP coding sequence, respectively, characterizes a “Swedish family” mutation. Similarly, the PSΔE9 mutation, identified as an exon 9 deletion mutation associated with familial AD, was observed.

A total of 20 C57 male mice (aged 5 months, weighing 25-30 g) were randomly assigned to groups A and B (10 mice/group). Additionally, 20 C57BL/6 male mice (specific pathogen-free [SPF] grade; Charles River, Zhejiang, China) were randomly allocated to groups C and D (10 mice/group). Subsequent to grouping, all mice underwent labeling. The mice were accommodated in an SPF-grade environment (4 mice per cage) with ad libitum access to food and water, maintaining a humidity range of 50%-70% at a room temperature of 20-25 °C. A one-week acclimatization period in an animal housing environment preceded the commencement of experiments, and all procedures adhered to ethical requirements concerning the use of experimental animals.

### 2.2 Main reagents and equipment

The reagents and equipment used in this study comprised of hematoxylin (Sigma, St Louis, MO, USA), eosin (Sigma), mTOR (Cell Signaling Technologies [CST], Beverly, MA, USA), p62 (Abcam, Cambridge, UK), PI3K (CST), LC3 (CST), Aβ1-42 (Proteintech, Wuhan, China), glyceraldehyde 3-phosphate dehydrogenase (Beyotime, Shanghai, China), 3,3′-diaminobenzidine color solutions (ZSGB-Bio, Beijing, China), Morris Water Maze (Shanghai Huaibei Equipment Co., Ltd., Shanghai, China), and an animal HBO chamber (Shanghai Tade Intelligent Technology Co., Ltd., Shanghai, China). Main reagents information has been listed in Supplementary Table 1.

### 2.3 General conditions of mice

The day of purchase was designated as day 0 (D0), and all animals underwent a one-week acclimatization period. Throughout this acclimatization period, the animals were provided with standard food and water ad libitum, and their weight was measured daily. Observations were made regarding the condition of their fur, food and water consumption, presence of bleeding and ulceration on toes and tails, level of activity, and mental status.

### 2.4 Preliminary Morris water maze assay

The preliminary Morris water maze assay was administered following the acclimatization and grouping periods, specifically on day 8 (D8). Prior to commencing the experiment, the Morris water maze was used for a 4-day orientation navigation training regimen. The objective of this training method was to assess the learning and memory capabilities of the mice, who were tasked with locating submerged platforms within the water, despite their aversion to it. Throughout the experiment, ambient conditions remained consistent, and the reference points remained unchanged. Each day, four quadrant tests were conducted, and the results were averaged. Following the orientation navigation assay, the platform was removed, and a spatial probe assay was conducted. The number of platform crossings and the duration of time spent in the target quadrant were meticulously recorded to analyze the search behavior exhibited by the mice.

### 2.5 HBO intervention

Following the preliminary Morris water maze assay, mice in groups A and C were subjected to normal feeding without any additional intervention, while mice in groups B and D were placed inside the animal HBO chamber daily at 9:00 am (Wang et al., 2022). The chamber underwent a gradual pressurization process with pure oxygen until reaching 0.20 MPa (2 atmosphere absolute [ATA]), followed by a slow depressurization, three subsequent washes with pure oxygen, and then a repeat pressurization to 0.20 MPa (2.0 ATA). Subsequently, the mice were exposed to oxygen inhalation for 60 minutes, and were removed from the chamber after a uniform 20-minute depressurization period. The HBO regimen containing two courses was performed and each course consists of 10 interventions lasting 1 day, with a 2-day interval after each intervention. The general condition of the mice was continuously monitored and meticulously recorded.

### 2.6 Morris water maze assay

Following the HBO treatment, mice were allowed a recovery period of 2 days before undergoing the Morris water maze assay, which included both spatial probe and orientation navigation assessments. The testing methodology remained consistent with the procedures described previously.

### 2.7 Hematoxylin-eosin (HE) staining was conducted to observe the morphology of hippocampal neurons

Following the completion of the tests, mice were euthanized, and brain tissue was collected following perfusion of the left ventricle. The obtained brain tissue was then divided into right and left cerebral hemispheres. Hippocampal tissues from one hemisphere were isolated and stored in a −80°C refrigerator. Brain tissues from the other hemisphere were fixed in 4% paraformaldehyde solution, subsequently sectioned, and subjected to staining, followed by morphological observation of hippocampal neurons using an inverted microscope.

### 2.8 Immunohistochemistry to detect Aβ42 and LC3 protein expression

The routinely sectioned brain tissues underwent deparaffinization and hydration, followed by antigen retrieval. Subsequently, 3% H2O2 was used to block and neutralize endogenous peroxidases. The tissues were then incubated with Aβ1-42 antibodies (dilution: 1:500) overnight at 4°C, followed by treatment with secondary antibodies (goat anti-rabbit/anti-mouse antibodies) at 37°C for 15 minutes. Color development was achieved using 3,3′-diaminobenzidine, followed by thorough rinsing with deionized water, hematoxylin staining, dehydration, clearing, and sealing. Finally, the tissues were subjected to observation and photographic documentation.

### 2.9 Western blot assay

Western blot assay was conducted as reported before (Zhang and Liu, 2013). The hippocampal tissues, stored in the −80°C refrigerator, were retrieved and homogenized. Subsequently, a western blot assay was conducted to assess the relative expression of LC3-II, as well as the expression levels of p62, PI3K, and mTOR proteins.

### 2.10 Statistical analysis

The data were processed using SPSS 20.0 statistical software, with measurement data expressed as mean ± standard deviation −(*X ± S)*. Intergroup comparisons were conducted using the *t*-test, and a significance level of *P* < 0.05 was deemed indicative of statistically significant differences.

## 3 Results

### 3.1 Changes in the general conditions of mice

Throughout the testing period, mice in each group exhibited normal mental activity, maintained a regular diet, and possessed smooth and glossy fur. Notably, there were no instances of mortality observed among the mice during the testing phase.

### 3.2 Effects of HBO on the cognitive function of mice

#### 3.2.1 Morris water maze assay results

**FIGURE 1:**
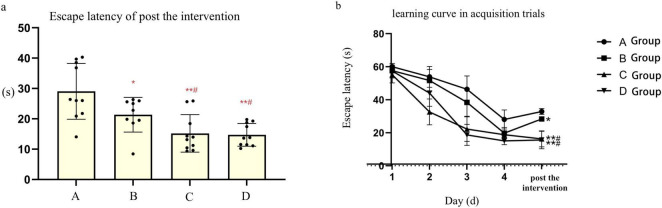
**(a)**, Point bar graphs for the difference of escape latency of post the after intervention. **(b)**, The learning curve inof acquisition trials of mice in each subgroups. *Compared to Group A, *P* < 0.05, ** Compared to Group A, *P* < 0.01, ^#^ Compared to Group B, *P* < 0.05

Prior to the experiment, mice in each group encountered difficulties in locating the submerged platform during the orientation navigation assay. However, following 4 days of training, the escape latency of mice gradually decreased across all groups. Specifically, the escape latency observed in groups A and B was longer compared to groups C and D (*t*-test, *P* < 0.05). Notably, there were no notable disparities in escape latency observed between groups A and B, nor between groups C and D. Following the HBO intervention, mice in group B exhibited markedly shorter escape latency and significantly prolonged dwell time in the target quadrant, with statistically significant differences observed compared to group A (*P* < 0.05) and groups C and D (*P* < 0.05). Conversely, no significant differences were noted in escape latency and dwell time in the target quadrant between groups C and D. Additionally, an increase in the number of platform crossings was observed in group B following the HBO intervention. However, the number of platform crossings did not significantly differ between groups A and B, nor between groups C and D. Furthermore, there were no notable discrepancies observed in the mean speed of mice among the various groups (refer to Table 1 and Figure 1).

**TABLE 1:** Comparisons of Morris water maze assay results.

Items		Group A	Group B	Group C	Group D
Escape latency (s)	Prior to intervention	28.07 ± 14.15	19.77 ± 8.29	18.84 ± 4.67**	15.13 ± 5.64**
	Post the intervention	29.05 ± 9.19	21.35 ± 5.69*	15.20 ± 6.19**^#^	14.71 ± 3.74**^#^
Dwell time in the target quadrant (s)	Prior to the intervention	29.91 ± 0.097	30.39 ± 1.61	34.77 ± 0.17**^#^	34.75 ± 0.19**^#^
	Post the intervention	29.89 ± 0.43	31.12 ± 1.35*	34.73 ± 0.46**^#^	34.60 ± 0.29**^#^
Number of platform crossings (n)	Prior to the intervention	1.90 ± 1.37	1.60 ± 1.51	4.10 ± 1.20**^#^	4.20 ± 1.23**^#^
	Post the intervention	1.90 ± 1.20	2.50 ± 2.85	4.30 ± 1.06**^#^	5.00 ± 1.41**^#^
Mean speed (mm/s)	Post the intervention	309.41 ± 70.36	309.41 ± 52.87	298.45 ± 60.93	338.83 ± 26.85

* *P* < 0.05,

** *P* < 0.01 compared with group A;

^#^ *P* < 0.05 compared with group B.

#### 3.2.2 Morphologic changes of hippocampal neurons in each group

**FIGURE 2:**
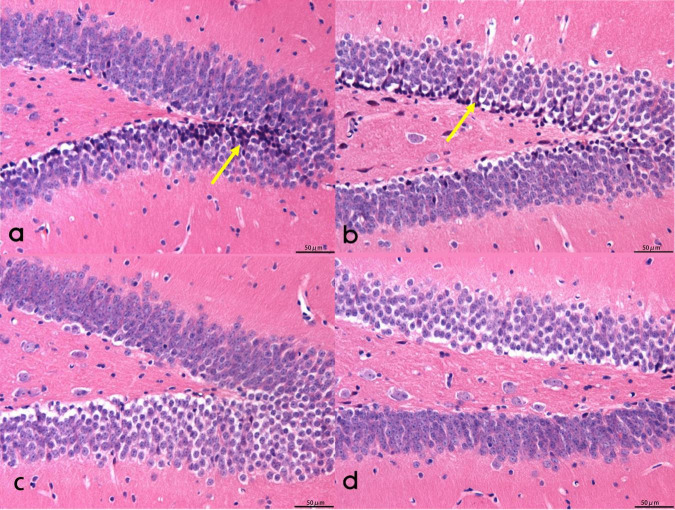
The morphology of hippocampal neurons in mice. **(a,b)** The neurons in the hippocampus of mice were not closely arranged, with pyknotic nuclei and polygonal nuclei, including a large number of neurofibrillary tangles. **(c)** Round nuclear cells in the hippocampus of mice. **(d)** Neurofibrillary tangle cells in the mouse hippocampus. Scale bar = 50 μm. Arrows for neurofibrillary tangles.

In groups A and B, hippocampal neurons exhibited a lack of tight arrangement, characterized by nuclear pyknosis and margination, along with a considerable presence of neurofibrillary tangles. Notably, the degree of nuclear pyknosis was reduced in group B when compared to group A. Conversely, hippocampal neurons in groups C and D displayed orderly arrangement and structural integrity, featuring rounded nuclei, clear nuclear staining, and normal nuclear morphology. While minimal nuclear pyknosis was observed at the periphery, and occasional neurofibrillary tangles were detected (Refer to Figure 2).

#### 3.2.3 Aβ_42_ expression in mouse hippocampal tissues in each group

**FIGURE 3:**
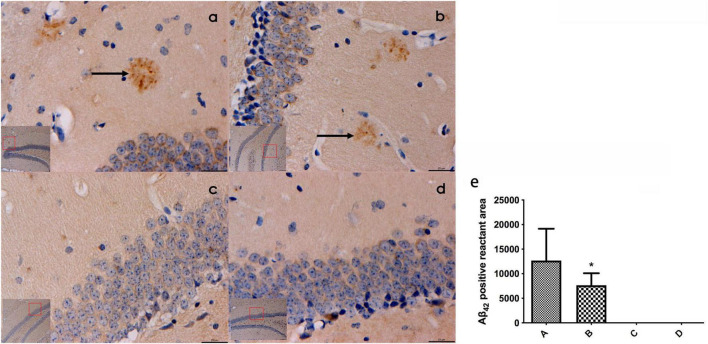
**(a–d)** Represented the expression of β42 in the hippocampal tissues of mice in the four groups (bar = 20 μm), and obvious patch-like Aβ42 positive reactants were found in the hippocampal tissues of mice in groups A and **(b)**, the area of Aβ42 positive reactants in mice in panel **(b)** was smaller than that in mice in panel **(a)** and **(e)** represented the comparison of the area of Aβ42 immunopositive reactants in the hippocampal tissues of mice in all groups. ← Indicates accumulation of plac-like Aβ42 immunopositive reactants, *compared to Group A, *P* < 0.05.

Prominent plaque-like Aβ_42_-positive deposits were identified within the hippocampal tissues of mice belonging to groups A and B. Notably, the area of Aβ_42_-positive deposits in group B was smaller compared to that in group A, with a statistically significant difference (*P* < 0.05). Conversely, no Aβ_42_-positive aggregates were observed in the hippocampal tissues of mice from groups C and D (Refer to Figure 3).

### 3.3 Effects of HBO on autophagy-related proteins in AD mice

#### 3.3.1 Expression of LC3-immunopositive reactants in mouse hippocampal tissues of each group detected by immunohistochemistry

**FIGURE 4:**
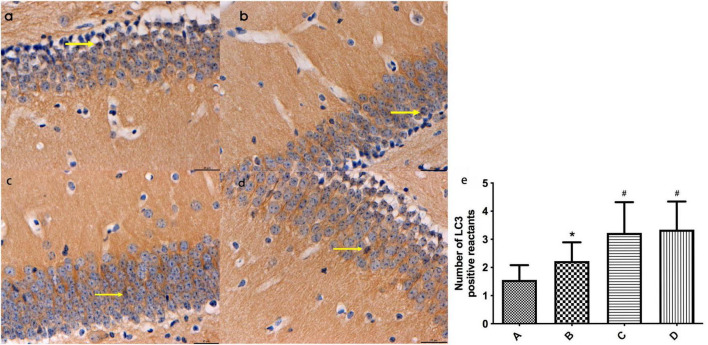
**(a-d)** Represent the distribution characteristics of LC3 immunoreactive positive substances in the hippocampal tissues of mice in the four groups (bar = 20 μm), and the LC3-positive reactants in the hippocampal tissues of mice in each group, respectively; **(e)** Represents the comparison of the distribution of LC3 immunoreactive positive substances in the hippocampal tissues of mice in the four groups, ← indicates LC3-positive reactants. *Compared to Group A, *P* < 0.05; #Compared to Group B, *P* < 0.05.

Based on the immunohistochemistry results, LC3-positive reactants were detected in the hippocampal tissues of mice across all groups. Notably, the intensity of LC3-positive staining in mouse hippocampal tissues was significantly higher in group B compared to group A (*P* < 0.05). Additionally, the levels of LC3-positive staining in mouse hippocampal tissues were notably elevated in groups C and D compared to groups A and B (*P* < 0.05). No significant difference was observed between groups C and D (Refer to Figure 4).

#### 3.3.2 mTOR, PI3K, p62, and LC3-II expression in hippocampal tissues of mice in each group

**FIGURE 5:**
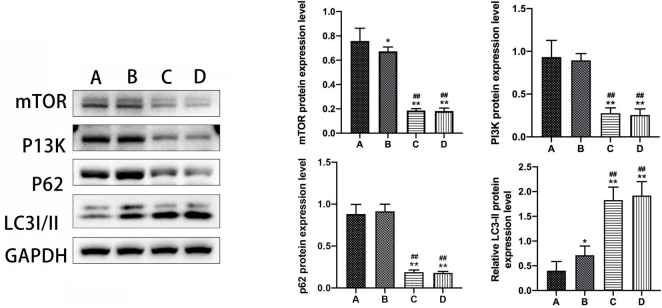
The expression levels of mTOR, PI3K, P62, and LC3-II in the hippocampal tissue of mice in subgroups. *Compared to Group A, *P* < 0.05; **compared to Group A, *P* < 0.01; # #Compared to Group B, *P* < 0.01.

The results from the western blot assay revealed that mTOR protein expression was significantly lower, while LC3-II protein expression was notably higher in group B compared to group A, with a statistically significant difference observed (*P* < 0.05). Furthermore, groups A and B exhibited significant changes in mTOR and LC3-II protein expression compared to groups C and D (*P* < 0.01). Conversely, no substantial difference was observed in the expression levels of PI3K and p62 proteins in mouse hippocampal tissues between groups B and A. Additionally, there were no discernible disparities noted in the expression levels of mTOR, PI3K, LC3-II, and p62 proteins in mouse hippocampal tissues between groups C and D (Refer to Figure 5).

## 4 Discussion

AD is a primary contributor to dementia in the elderly population, marked by progressive memory impairment as its main symptom, often accompanied by the gradual onset of language deficits, comprehension difficulties, impaired cognitive functions such as arithmetic and orientation, as well as notable changes in personality and behavioral patterns, culminating in an inability to perform self-care activities. The manifestation of personality changes is frequently observed at the initial consultation phase. AD typically follows an insidious onset and exhibits a rapid progression, lacking specific clinical treatments at present. HBO, a pivotal non-pharmacological intervention widely used in clinical settings, is characterized by exposure to oxygen at partial pressures greater than 1.0 ATA in environments surpassing atmospheric pressure levels. HBO holds promise in enhancing cognitive function among patients with AD. Its therapeutic mechanisms encompass the mitigation of oxygen free radicals, augmentation of oxygen diffusion distances and cerebral oxygenation levels, facilitation of cellular proliferation and functionality, acceleration of capillary regeneration, and optimization of cerebral blood flow, thereby promoting neural function and exerting beneficial conditioning and therapeutic effects on the organism. Furthermore, HBO demonstrates the capacity to mitigate cell apoptosis, suppress inflammatory responses, and stimulate stem cell activation (Schottlender et al., 2021). Therefore, HBO finds extensive application in the clinical management of conditions such as CO poisoning, ischemic-hypoxic disorders, stroke, cerebral edema, neurodegenerative conditions, spinal cord injuries, traumatic pathologies, and inflammatory diseases.

In this study, the Morris water maze assay was used to assess the orientation navigation and spatial probe capabilities of model mice after the administration of HBO intervention. The findings revealed that mice with AD subjected to HBO intervention exhibited shorter escape latencies and prolonged dwell times in the target quadrant compared to mice with AD without intervention, indicating an enhancement in cognitive functions facilitated by HBO. Additionally, histological analyses via HE staining and immunohistochemistry of mouse hippocampal tissues demonstrated a reduction in neurofibrillary tangles and Aβ deposits among mice with AD following HBO intervention, indicative of the potential of HBO to mitigate Aβ deposits and neurofibrillary tangles, thereby ameliorating cognitive function in AD mice. Corroborating evidence from Shapira et al. revealed the efficacy of HBO treatment in AD management, with observed suppression of hypoxia, reduction in amyloid deposition, and tau phosphorylation levels in mice with AD, leading to an improvement in cognitive function (Shapira et al., 2018). Similarly, Choi et al. reported that 4 or 8 weeks of HBO treatment attenuated spatial learning and memory deficits, alongside decreased Aβ deposition and neuroinflammatory plaque formation in the cortex and hippocampus of mice (Choi et al., 2019).

Cellular autophagy constitutes a fundamental physiological process through which cells uphold intracellular homeostasis by dismantling and recycling long-lived proteins and dysfunctional organelles, thereby functioning as an inherent self-regulatory mechanism. This metabolic pathway, operating through lysosomal degradation mechanisms, embodies a distinctive biological phenomenon within eukaryotic cells and plays a pivotal role in cell survival, differentiation, development, and overall homeostasis. The mammalian nervous system, particularly neurons, heavily relies on autophagy to mitigate the accumulation of large insoluble protein aggregates, thereby preserving cellular equilibrium. Sunshine et al. conducted an analysis to elucidate the therapeutic mechanism of HBO treatment in mice with spinal cord injury (SCI) (Sunshine et al., 2023). Their findings underscored that HBO treatment further enhanced neuron autophagy in SCI-affected mice, thereby expediting cell repair and reconstruction, and reducing the duration of the disease. These findings suggest that HBO is an effective intervention for SCI, with the enhancement of neuron autophagy being a potential mechanism through which HBO exerts its therapeutic effects on SCI.

Studies have reported a close association between AD progression and autophagy, where levels of Aβ, neuronal survival, apoptosis, and behavioral memory recovery are intertwined with autophagic processes. Dysfunction in autolysosomes has been observed in the early-stage of patients with AD (de la Cueva et al., 2022; Fang et al., 2019; Mary et al., 2023). Autopsies of patients with AD have revealed the presence of numerous autophagic vacuoles containing Aβ1-40, Aβ1-42, APP, and secretases in brain tissues (Yu et al., 2004). Aberrations in autophagosome transport or lysosomal degradation efficiency contribute to autophagosome accumulation and subsequent formation of SPs due to excessive Aβ deposition (Lee et al., 2022; Schmukler and Pinkas-Kramarski, 2020). Previous research has indicated that over-phosphorylated tau protein is cleared through the autophagy-lysosomal pathway with the aid of the autophagy receptor NDP52. Conversely, inhibition of autophagic activity facilitates tau protein phosphorylation (Hamano et al., 2021; Jo et al., 2014). The regulation of autophagy involves various pathways, including the AMPK and mTOR pathways. mTOR, a key negative regulator of autophagy, is implicated in AD pathology by suppressing autophagic function and diminishing Aβ clearance (Shi et al., 2022; Yang et al., 2023). mTOR influences Aβ production or clearance through multiple pathways, including PI3K, AKT, GSK-3, AMPK, and IGF-1 pathways. Furthermore, mTOR activation contributes to abnormal hyperphosphorylation of tau (Zhang et al., 2009; Lai et al., 2022). Rapamycin, an mTOR inhibitor, has been shown to ameliorate cognitive deficits and inhibit pathology associated with amyloid plaques and neurofibrillary tangles by enhancing cell autophagy.

In the current investigation, the observed upregulation of LC3-II expression in mouse hippocampal tissues following HBO intervention among mice with AD indicates a potential influence of HBO on AD via autophagy-related genes. Additionally, the downregulation of mTOR protein expression in AD mice subjected to HBO intervention compared to control AD mice indicates that the mTOR pathway is activated to induce autophagy during HBO treatment in AD mice. In other words, HBO may exert therapeutic effects on AD by activating the mTOR pathway-mediated autophagy.

Autophagy is a key to cellular homeostasis. HBO activates a number of autophagy-related signaling pathways, such as mTOR signaling pathway, by affecting the intracellular oxygen pressure. Activation of mTOR pathway can inhibit autophagy, while HBO can inhibit mTOR activation. Hence, HBO can rescue autophagy, promote the formation of autophagosomes and the fusion of autolysosomes, leading to the removal of AD-related pathological proteins and improving neuronal function. In addition, oxidative stress associated with HBO is also related to autophagy. There are higher levels of oxidative stress in the brain of AD mice, which can lead to cell damage and dysfunction. Enhanced activity of antioxidant enzymes and reduction of reactive oxygen species also contribute to rescue autophagy. At the same time, moderate oxidative stress can be used as a stimulus signal to induce cells to initiate autophagy to remove damaged components and maintain cell homeostasis. On the other hand, mitochondria play an important role in the pathogenesis of AD. HBO can improve mitochondrial respiratory function and energy production efficiency, improve mitochondrial function, thereby promoting the survival and functional recovery of neurons, recovering the cognitive function of AD mice.

In conclusion, HBO demonstrates efficacy in enhancing cognitive function in mice with AD, potentially mediated through the regulation of cellular autophagy. These findings contribute to the theoretical underpinning for the clinical use of HBO as a therapeutic modality for AD treatment. The research of this field is still in the continuous exploration and development stage, more investigations are needed to initiate for clarifying the association between autophagy and AD. Delving deeper into the pathways implicated in the interplay between autophagy and AD could unveil novel therapeutic avenues for patients diagnosed with AD.

## Data Availability

The raw data supporting the conclusions of this article will be made available by the authors, without undue reservation.
