# Supplementary material for: Enhancement of cognitive function in mice with Alzheimer’s disease through hyperbaric oxygen-induced activation of cellular autophagy
Source: Front Aging Neurosci. 2024 Sep 25;16:1418081. doi: 10.3389/fnagi.2024.1418081 (PMC11461206; doi:10.3389/fnagi.2024.1418081)
Supplement: Supplementary file 1 [file Table_1.DOCX]

| **Supplementary table 1: Main reagents information** | | | |
| --- | --- | --- | --- |
| Reagent name | Productor | Catalog number | Dilution ratio |
| mTOR | CST | 2983 | 1: 2000 |
| p62 | abcam | ab109012 | 1:1000 |
| PI3Kinase | CST | 4292 | 1:1000 |
| LC3A/B | CST | 12741 | 1:1000 |
| GAPDH | Beyotime | AF0006 | 1:1000 |
| Goat anti-Rabbit IgG | Multi Science | GAR0072 | 1:5000 |
| Goat anti-Mouse IgG | Multi Science | GAM007 | 1:5000 |
